# Macro- and micronutrient intakes in picky eaters: a cause for concern?^1^,^2^,^3^

**DOI:** 10.3945/ajcn.116.137356

**Published:** 2016-11-09

**Authors:** Caroline M Taylor, Kate Northstone, Susan M Wernimont, Pauline M Emmett

**Affiliations:** 4 Centre for Child and Adolescent Health, School of Social and Community Medicine, University of Bristol, Bristol, United Kingdom;; 5 National Institute for Health Research Collaboration for Leadership in Applied Health Research and Care West, Bristol, United Kingdom; and; 6 Nestlé Nutrition, King of Prussia, PA

**Keywords:** ALSPAC, picky eating, macronutrients, micronutrients, antioxidants, meat, vegetables, fruits

## Abstract

**Background:** Picky eating (PE) is characterized by an unwillingness to eat certain foods and by strong food preferences. PE may result in lower intakes of energy and nutrients, which may compromise health.

**Objectives:** We quantified nutrient and food group intakes in children identified as picky eaters or nonpicky eaters and compared intakes between groups and with United Kingdom reference nutrient intakes.

**Design:** PE was identified in an observational cohort (Avon Longitudinal Study of Parents and Children) from questionnaires administered when children were aged 2, 3, 4.5, and 5.5 y. Dietary intake was assessed at 3.5 and 7.5 y with a 3-d food record. The dietary assessment at 3.5 y compared picky eaters with nonpicky eaters identified at age 3 y, and the assessment at 7.5 y compared longitudinally defined PE groups.

**Results:** Picky eaters aged 3 y had lower mean carotene, iron, and zinc intakes than nonpicky eaters. There were similar differences between the longitudinally defined PE groups. Iron and zinc intakes were most likely to be below recommended amounts, with free sugar intake much higher than recommended. There were no significant differences in energy intakes between the groups, and intakes were adequate relative to estimated average requirements. Nutrient differences were explained by lower intakes of meat, fish, vegetables, and fruits in picky eaters than in nonpicky eaters. There were higher intakes of sugary foods and drinks in older picky eaters.

**Conclusions:** PE did not result in compromised macronutrient intakes, although intakes of zinc and iron were more likely to be below recommendations for picky eaters than for nonpicky eaters. Emphasis should be placed on allaying parental concerns about picky eaters being prone to inadequate nutrient intakes and on encouraging all parents to extend their child’s diet to include more nutrient-rich items, especially fruits and vegetables, and less nutrient-poor sugary foods.

## INTRODUCTION

There is little consensus on the definition of picky eating, although it generally includes the rejection of specific familiar and unfamiliar foods (1, 2). Picky eating can lead to a reduction in dietary variety and consequently an unhealthy or possibly inadequate diet (3–5), with picky eaters eating a smaller range of food items than nonpicky eaters and having lower diversity and variety scores (3–6). In turn, this can result in being underweight and having poor growth (7–16) or in being overweight (17), having gastrointestinal disorders (18), or developing eating disorders (19).

Several studies have focused on the association of picky eating with particular food groups. A common theme is a reduced intake of vegetables, and to a lesser extent fruits, in picky eaters compared with nonpicky eaters (6, 15, 16, 20–26). Reductions in the consumption of whole-grain products, fish and seafood, meat, and unsweetened cereals and increases in savory snacks and confectionary cereals and French fries in picky eaters compared with nonpicky eaters have also been noted (22, 23). A recent study found that picky eaters ate a lower total number of foods (25).

Although it seems to be well established that picky eating results in a difference in the intake of some micronutrients (4, 15, 16, 24), it has not been clearly established whether this difference is meaningful relative to recommended dietary guidelines. Some studies have reported comparisons with recommended daily intakes and have found intakes of several nutrients below recommended amounts (8, 27). However, many studies lack a comparator group of nonpicky eaters and are thus difficult to interpret. There are very few data on longitudinal dietary intakes, and there is a pressing need for studies that include a comparison both with a nonpicky eating group and with appropriate recommended dietary intakes. This would ultimately enable evaluations of the effect of picky eating on dietary intakes and on health outcomes and facilitate the development of advice to parents and health professionals on how to manage picky eating. We have already identified groups of picky eaters within a longitudinal cohort of children from the United Kingdom ALSPAC^7^ (Avon Longitudinal Study of Parents and Children) cohort (2) in whom picky eating was measured at 4 time points between the ages of 2 and 5.5 y. As reported previously by Taylor et al. (2), the prevalence of picky eating in this population was 9.7% in children aged 2 y, 14.7% in those aged 3 y, 14.2% in those aged 4.5 y, and 11.8% in those aged 5.5 y. We used these groups of picky eaters and a comparator group of nonpicky eaters in this study to compare nutrient intakes cross-sectionally in children aged 3 y (the age with the greatest prevalence of picky eating in this study group). In addition, we define groups of picky eaters (never, high or low, early-onset or late-onset, persistent or not persistent) longitudinally over the 4 time points and compare dietary intakes at the age of 7 y between groups. The dietary intakes of the groups are also compared with appropriate recommended reference values for macro- and micronutrients.

## METHODS

ALSPAC is a longitudinal population-based study investigating environmental and genetic influences on the health, behavior, and development of children. It has covered diet extensively and collected questionnaire data from parents about feeding their child.

All pregnant women in the former Avon Health Authority in South West England with an expected delivery date between 1 April 1991 and 31 December 1992 were eligible for the study; 14,541 pregnant women were enrolled, resulting in a cohort of 14,062 live births, with 13,988 alive at the age of 1 y (28). The social and demographic characteristics of this cohort were similar to those found in United Kingdom national census surveys (29). Further details of ALSPAC are available at http://www.bris.ac.uk/alspac, and the study website contains details of all the data that are available through a fully searchable data dictionary (http://www.bris.ac.uk/alspac/researchers/data-access/data-dictionary). Ethics approval was obtained from the ALSPAC Ethics and Law Committee and the local research ethics committees.

### Defining picky eating in the ALSPAC cohort

**FIGURE 1:**
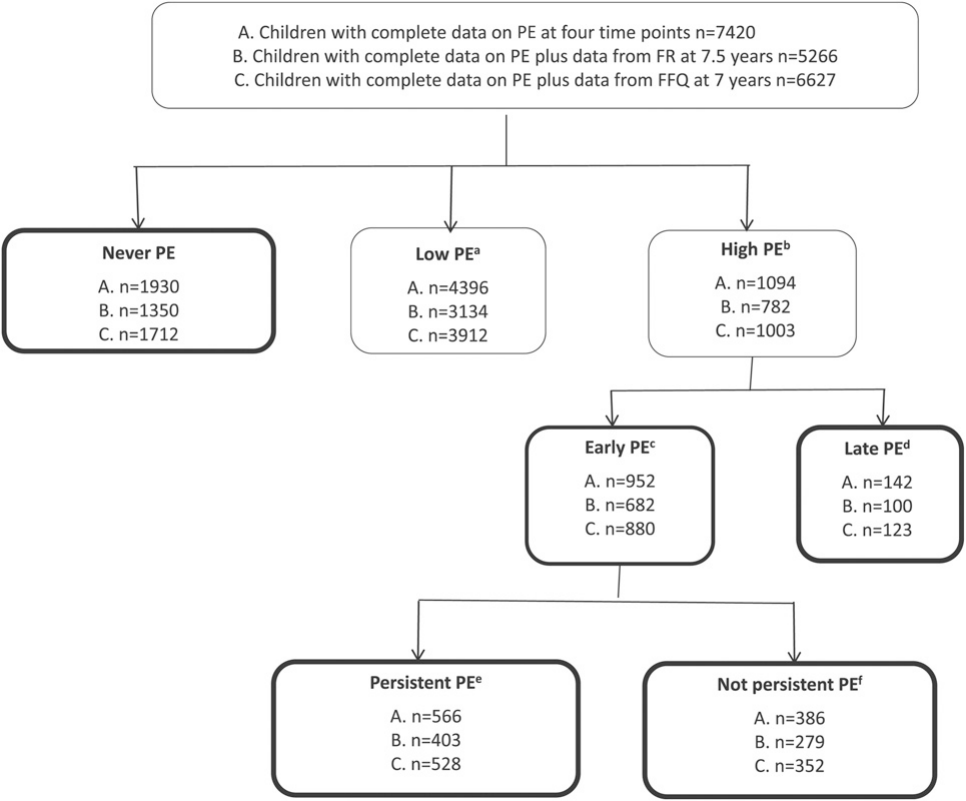
Flowchart of PE definitions for the analysis of longitudinal picky eating. ^a^Low PE, score of 1 at all time points or 0 or 1 at 3 points and 2 at any 1 time point; ^b^high PE, score of 2 at ≥2 time points; ^c^early PE, in high group and score of 2 at first and/or second time point; ^d^late PE, in high group and score of 2 for the first time at third time point; ^e^persistent, in early group and a score of two 3 or 4 times; and ^f^not persistent, in early group and score of 2 twice. FFQ, food-frequency questionnaire; FR, food record; PE, picky eating.

The primary caregiver (usually the mother) received postal self-completion questionnaires when their child was aged 2, 3, 4.5, and 5.5 y. The questionnaires are available from the study website (http://www.bristol.ac.uk/alspac/researchers/questionnaires). Singletons only (of any birth order) were selected for all phenotype definitions. The following question, which was similar to those used in several other studies (30–33), was asked at 4 time points up to the age of 5 y (2, 3, 4.5, and 5.5 y): “Does your child have definite likes and dislikes as far as food is concerned?” There were three possible responses: no; yes, quite choosy; and yes, very choosy. The responses were scored 0, 1, or 2, respectively. For cross-sectional definitions of picky eating at each time point, picky eaters were defined as those who scored 2 and nonpicky eaters as those who scored 0. Children who scored 1 were considered to be somewhat picky. The longitudinal prevalence of picky eating across children aged 2–5.5 y was calculated from the number of children who scored 2 at ≥2 time points. Early-onset picky eating was defined as the first report of picky eating (score 2) being at either 2 or 3 y, and late-onset picky eating was defined as the first report of picky eating being at 4.5 or 5.5 y. Persistent picky eating was defined as being in the early-onset group and scoring a 2 at 3 or 4 time points (**Figure 1**).

### Dietary assessment

#### Food records

A convenience subsample of 10% of the ALSPAC cohort was invited to a research clinic when the children were aged 3.5 y. Parents were mailed a structured diary beforehand to record all the foods and drinks that the child consumed over 3 individual days (1 weekend day and 2 weekdays) in household measures. The food records (FRs) were checked with the parents in the clinic and then used to calculate daily mean nutrient intakes for each child, as described by Emmett et al. (34). In brief, the records were coded and analyzed with the use of an in-house nutrient analysis program based on the fifth edition of McCance and Widdowson’s food tables and supplements to the tables [cited in Emmett et al. (34)]. Free sugars were all monosaccharides and disaccharides added to foods by the manufacturer, cook, or consumer plus sugars naturally present in honey, syrups, fruit juices, and fruit juice concentrates. A similar method was used when the children were aged 7.5 y, and the entire ALSPAC cohort of children was invited to attend.

#### Food-frequency questionnaire

Parental-completion questionnaires were mailed out when children were aged 3 and 6.75 y (rounded to 7 y). Each questionnaire contained a range of questions about the child with a section about eating, including a full food-frequency questionnaire (FFQ). The list of foods covered by the FFQ can be found in North and Emmett (35). The FFQ was used to estimate daily intakes of nutrients and amounts of foods consumed as described in detail by Rogers and Emmett (36).

### Statistical analysis

Statistical analysis was carried out with SPSS version 21 (IBM). ANOVA was used for 2 main analyses in which data from the FR are presented, with FFQ data as supplemental tables to confirm the results from the FR: *1*) cross-sectional analysis of dietary intakes of macro- and micronutrients at 3.5 y (FR) and 3 y (FFQ) by picky eating scores at 3 y and *2*) analysis of intakes at 7.5 y (FR) and 7 y (FFQ) by longitudinal picky eating type from 2 to 5.5 y. Statistical comparisons were made between the different categories [ANOVA with multiple comparisons (Bonferroni correction); superscript letters were generated with a computer program (37)]. Energy intakes were calculated separately for males and females to enable a comparison with sex-specific recommendations on estimated average requirements (38). The percentage of children with intakes below the United Kingdom–recommended reference nutrient intake (RNI) and the lower RNI (LRNI) (the amount at which intake is almost certainly inadequate for an individual) was calculated for micronutrients for which differences between picky eating groups were evident (39). Mean weights consumed of each food group (**Supplemental Text**) were compared between picky eating groups; only those with statistically significant differences in FR intakes between the picky eating groups are presented.

## RESULTS

### Cross-sectional analysis at 3.5 y: macronutrients

Children who were picky eaters at the age of 3 y consumed similar amounts of energy at the age of 3.5 y as did children who were not picky eaters, suggesting that identifying picky eaters was not simply a proxy for lower appetite. The mean daily energy intake for all 3 groups of children exceeded the current United Kingdom–recommended intakes for children aged 3 y (38) (**Table 1**). Although there are no specific recommendations for children, the percentages of energy from protein, carbohydrates, and fat for all 3 groups were similar to the percentages recommended by the United Kingdom Committee on Medical Aspects of Food Policy for adults of 15%, 50%, and 35% energy, respectively (39) (data not shown). The percentage of energy from free sugars in all 3 groups was greater than the United Kingdom and WHO recommendations of ≤10% energy [39, 40; United Kingdom guideline revised to <5% in 2015 (41)]. Only 11.8% of picky eaters and 11.5% of nonpicky eaters consumed <10% energy as free sugars. There was a slightly lower protein intake (−3.2 g/d) for picky eaters than nonpicky eaters (*P* = 0.021), but no child in any group had an intake below the United Kingdom estimated average requirement (39). Data from the FFQ are shown in **Supplemental Table 1**.

**TABLE 1:** Macro- and micronutrient intakes, assessed by food record at 3.5 y, by picky eating score at age 3 y in a subsample of ALSPAC children^1^

		Picky eating score (cross-sectional)		
	Recommendation, EAR, or RNI^2^	0	1	2
*n*		364	320	131
Macronutrients				
Energy, kJ/d	—	5704 (5592, 5817)	5650 (5532, 5769)	5631 (5451, 5811)
Males	4900^3^	5812 (5660, 5965)	5841 (5671, 6011)	5803 (5572, 6033)
Females	4500^3^	5555 (5391, 5719)	5436 (5278, 5595)	5387 (5104, 5670)
Protein, g/d	14.5^4^	47.9 (46.7, 49.1)^a^	46.1 (44.8, 47.4)^a,b^	44.7 (42.7, 46.7)^b^
Carbohydrates, g/d	—	178 (174, 182)	175 (171, 179)	176 (168, 179)
Fat, g/d	—	55.2 (53.8, 56.6)	55.9 (54.5, 57.4)	56.9 (54.4, 59.3)
Free sugars, % energy	<10^5^	16.0 (15.4, 16.6)	15.8 (15.1, 16.4)	16.6 (15.6, 17.5)
Micronutrients				
Vitamin A				
Retinol, μg/d	—	370 (331, 409)	355 (329, 381)	365 (332, 399)
Carotene,^6^ μg/d	—	1534 (1413, 1655)^a^	1379 (1270, 1489)^a,b^	1152 (952, 1354)^b^
REs, μg REs/d	400^4^	626 (581, 670)	585 (552, 618)	558 (510, 605)
Thiamin, mg/d	0.4 mg/4.2 MJ^4^	1.00 (0.98, 1.03)	0.98 (0.92, 1.03)	0.93 (0.89, 0.97)
Riboflavin, mg/d	0.6^4^	1.44 (1.41, 1.49)	1.45 (1.40, 1.51)	1.53 (1.44, 1.62)
Niacin, mg NEs/d	6.6 mg NEs/4.2 MJ^4^	21.2 (20.7, 21.8)^a^	20.3 (19.7, 20.9)^a,b^	19.4 (18.6, 20.2)^b^
Vitamin B-6, mg/d	15 μg/g protein^4^	1.35 (1.31, 1.39)^a^	1.30 (1.26, 1.34)^a,b^	1.24 (1.17, 1.29)^b^
Vitamin B-12, μg/d	0.5^4^	3.14 (3.01, 3.27)	3.18 (3.02, 3.33)	3.10 (2.86, 3.34)
Folate, μg/d	70^4^	154.4 (149.8, 159.0)	149.7 (144.7, 154.7)	146.1 (138.1, 154.1)
Vitamin C, mg/d	30^4^	55.2 (51.1, 59.3)	53.8 (49.7, 57.9)	54.2 (46.0, 62.3)
Vitamin D, μg/d	7^4^	1.84 (1.74, 1.95)	1.75 (1.59, 1.91)	1.73 (1.38, 2.08)
Vitamin E, mg/d	—	6.16 (5.91, 6.42)	6.07 (5.81, 6.34)	5.71 (5.28, 6.14)
Calcium, mg/d	350^4^	754 (728, 780)	774 (740, 807)	796 (740, 853)
Iron, mg/d	6.9^4^	6.5 (6.3, 6.6)^a^	6.2 (6.0, 6.4)^a,b^	5.9 (5.5, 6.2)^b^
Zinc, mg/d	5.0^4^	5.3 (5.2, 5.5)^a^	5.1 (4.9, 5.2)^a,b^	4.9 (4.6, 5.1)^b^
Selenium, μg/d	15^4^	43.6 (42.2, 45.0)^a^	42.0 (40.4, 43.5)^a^	38.3 (36.2, 40.3)^b^
Iodine, μg/d	70^4^	149.2 (141.6, 156.9)	152.7 (143.4, 162.1)	161.2 (144.1, 178.4)

1 Values are means (95% CIs) unless otherwise indicated. Values in the same row without a common superscript letter are significantly different, *P* < 0.05; rows with no letters have no significant differences between values [ANOVA with multiple comparisons (Bonferroni)]. Picky eating scores were assessed with the use of a questionnaire and were defined as follows: 0, not choosy; 1, quite choosy; and 2, very choosy. ALSPAC, Avon Longitudinal Study of Parents and Children; EAR, estimated average requirement; NE, niacin equivalent; RE, retinol equivalent; RNI, reference nutrient intake.

2 Intake above RNI will almost certainly be adequate (2 notional SDs above EAR).

3 From reference 38.

4 From reference 39.

5 From references 39 and 40 [United Kingdom guideline revised to <5% in 2015 (41)].

6 Carotene is in the form of β-carotene equivalents: sum of β-carotene and half the amounts of α-carotene and α- and β-cryptoxanthins.

### Cross-sectional analysis at 3.5 y: micronutrients

Picky eaters had lower mean intakes of several micronutrients than nonpicky eaters at the age of 3.5 y (carotene 25% lower, niacin and vitamin B-6 both 8% lower, iron and zinc both 9% lower, selenium 12% lower) (Table 1). However, the percentages of children with intakes below the United Kingdom RNI or LRNI were very low or zero for niacin, vitamin B-6, and selenium (**Table 2**). For iron and zinc, one-half to three-fourths of all 3 groups of children had intakes below the RNI, with picky eaters more likely than nonpicky eaters to be in this category. The proportion of children with intakes below the LRNI for iron and zinc was not different between picky eating groups (Table 2). There are no recommendations for carotene intake separately from retinol. Data from the FFQ are shown in Supplemental Table 1 and** Supplemental Table 2**.

**TABLE 2:** Comparisons of micronutrient intakes, assessed by food record at 3.5 y of age, with United Kingdom RNI and LRNI by picky eating score at 3 y (cross-sectional) in a subsample of ALSPAC children^1^

		Children below recommended intake, %		
	United Kingdom RNI or LNRI	PE score 0	PE score 1	PE score 2
*n*		364	320	131
United Kingdom RNI^2^ (39)				
Niacin	6.6 mg NEs/4.2 MJ	0.0	0.0	0.0
Vitamin B-6	15 μg/g protein	0.3	0.6	0.0
Iron	6.9 mg/d	65.4^a^	70.7^a,b^	78.6^b^
Zinc	5 mg/d	47.0	53.8	58.8
Selenium	15 μg/d	0.3	1.3	1.5
United Kingdom LRNI^3^ (39)				
Niacin	4.4 mg NEs/4.2 MJ	0.0	0.0	0.0
Vitamin B-6	11 μg/g protein	0.0	0.0	0.0
Iron	3.7 mg/d	4.1	5.3	6.1
Zinc	3 mg/d	3.0	5.6	5.3
Selenium	7 μg/d	0.0	0.0	0.0

1 Bonferroni correction (*z* tests) was used for column proportion comparisons. Values in the same row without a common superscript letter are significantly different, *P* < 0.05; rows with no letters have no significant differences between values. PE scores were assessed with the use of a questionnaire for children aged 3 y and were defined as follows: 0, not choosy; 1, quite choosy; and 2, very choosy. ALSPAC, Avon Longitudinal Study of Parents and Children; EAR, estimated average requirement; LRNI, lower reference nutrient intake; NE, niacin equivalent; PE, picky eating; RNI, reference nutrient intake.

2 Intake above which will almost certainly be adequate (2 notional SDs above EAR).

3 Intake below which will almost certainly be inadequate for most individuals (2 notional SDs below EAR).

### Longitudinally defined picky eating types and diet at 7.5 y: macronutrients

Mean energy intakes at 7.5 y were similar between the picky eating groups and exceeded the United Kingdom–recommended intake for children aged 7 y (38) (**Table 3**). Mean protein intake was greatest in the never group and 8% lower in the persistent (*P* < 0.001) and 11% lower in the late-onset groups (*P* < 0.001), but none of the children had an inadequate intake (39). Mean intakes of free sugars as percentage of energy were higher in all the picky eating groups than in the never group, and mean intakes in all groups were above the recommended upper limit of 10% energy (39, 40). Again, the persistent and late-onset groups showed the largest differences compared with the never group (13% and 14% higher, respectively; both *P* < 0.001). Data from the FFQ are shown in **Supplemental Table 3**.

**TABLE 3:** Macro- and micronutrient intakes, assessed by food record at 7.5 y of age, by longitudinally defined picky eating types at ages 2–5.5 y in a subsample of ALSPAC children^1^

		Picky eating type				
				High		
				Early onset		
	Recommendation, EAR, or RNI^2^	Never	Low	Nonpersistent	Persistent	Late onset
*n*		1350	3134	279	403	100
Macronutrients						
Energy, kJ/d	—	7239 (7169, 7308)	7193 (7148, 7238)	7148 (6978, 7318)	7182 (7058, 7305)	6967 (6709, 7224)
Males	6900^3^	7495 (7397, 7592)	7473 (7409, 7536)	7552 (7324, 7780)	7377 (7213, 7541)	7403 (7022, 7786)
Females	6400^3^	6972 (6877, 7066)	6913 (6852, 6973)	6665 (6433, 6897)	6944 (6764, 7124)	6547 (6230, 6863)
Protein, g/d	28.3^4^	57.8 (57.1, 58.4)^a^	55.4 (55.0, 55.9)^b^	55.0 (53.4, 56.5)^b.c^	53.0 (51.8, 54.2)^c^	51.3 (48.7, 53.9)^c^
Carbohydrates, g/d	—	229 (227, 232)	231 (230, 232)	229 (223, 234)	232 (237, 236)	227 (219, 236)
Fat, g/d	—	70.0 (69.1, 70.9)	69.0 (68.4, 69.6)	69.2 (67.2, 71.3)	69.7 (68.1, 71.3)	66.6 (63.5, 69.7)
Free sugar, % energy	<10^5^	16.7 (16.4, 17.0)^a^	17.8 (17.6, 18.0)^b^	17.8 (17.1, 18.4)^a,b,c^	18.8 (18.2, 19.4)^c^	19.1 (18.1, 20.0)^b,c^
Micronutrients						
Vitamin A						
Retinol, μg/d	—	390 (366, 414)	358 (347, 369)	409 (337, 481)	369 (336, 401)	297 (270, 322)
Carotene,^6^ μg/d	—	2000 (1930, 2070)^a^	1888 (1840, 1937)^a^	1971 (1785, 2158)^a,b^	1678 (1503, 1852)^b,c^	1477 (1198, 1755)^c^
REs, μg/d	500^4^	723 (696, 750)^a^	673 (659, 686)^b^	738 (660, 815)^a,b^	649 (604, 692)^b,c^	543 (487, 598)^c^
Thiamin, mg/d	0.4 mg/4.2 MJ^4^	1.5 (1.5, 1.6)^a^	1.4 (1.4, 1.5)^b^	1.5 (1.4, 1.6)^a,b^	1.5 (1.4, 1.6)^a,b^	1.5 (1.2, 1.7)^a,b^
Riboflavin, mg/d	1.0^4^	1.7 (1.6, 1.7)^a,b^	1.6 (1.6, 1.7)^a^	1.7 (1.6, 1.7)^a,b^	1.8 (1.7, 1.8)^a,b^	1.6 (1.5, 1.8)^a,b^
Niacin, mg NEs/d	6.6 NEs/4.2 MJ^4^	27.0 (26.7, 27.3)^a^	26.2 (26.0, 26.5)^b^	26.1 (25.4, 26.8)^a,b^	25.4 (24.8, 26.0)^b^	24.9 (23.5, 26.4)^b^
Vitamin B-6, mg/d	15 μg/g protein^4^	1.8 (1.7, 1.8)	1.8 (1.7, 1.8)	1.8 (1.7, 1.8)	1.7 (1.7, 1.8)	1.7 (1.6, 1.8)
Vitamin B-12, μg/d	1.0^4^	1.9 (3.7, 3.9)^a^	1.7 (3.6, 3.7)^b^	1.9 (3.5, 4.0)^a,b^	1.8 (3.5, 3.8)^a,b^	2.0 (3.3, 4.1)^a,b^
Folate, μg/d	150^4^	203 (200, 206)	198 (196, 200)	195 (187, 203)	195 (188, 202)	186 (172, 199)
Vitamin C, mg/d	30^4^	81.9 (78.9, 84.9)	80.9 (79.0, 82.9)	79.9 (73.2, 86.6)	77.1 (70.5, 83.7)	78.4 (67.1, 89.7)
Vitamin D, μg/d	0^4^	2.5 (2.4, 2.5)^a^	2.4 (2.4, 2.4)^a,b^	2.4 (2.2, 2.6)^a,b,c^	2.2 (2.1, 2.3)^c^	2.1 (1.9, 2.3)^b,c^
Vitamin E, mg/d	—	8.2 (8.0, 8.4)	8.1 (8.0, 8.2)	8.1 (7.7, 8.5)	8.0 (7.6, 8.3)	7.8 (7.2, 8.4)
Calcium, mg/d	550^4^	804 (790, 818)	786 (776, 795)	788 (754, 822)	826 (797, 854)	769 (708, 830)
Iron, mg/d	8.7^4^	8.6 (8.5, 8.7)^a^	8.3 (8.3, 8.4)^b^	8.2 (7.9, 8.4)^b^	8.2 (7.9, 8.4)^b^	7.9 (7.4, 8.3)^b^
Zinc, mg/d	7.0^4^	6.3 (6.2, 6.4)^a^	6.0 (6.0, 6.1)^b^	5.9 (5.7, 6.1)^b,c^	5.9 (5.7, 6.0)^b,c^	5.4 (5.9, 5.7)^c^
Selenium, μg/d	30^4^	54.1 (53.2, 54.9)^a^	52.4 (51.8, 53.0)^b^	49.8 (47.8, 51.8)^b^	52.4 (48.7, 52.1)^b^	49.2 (45.4, 53.0)^a,b^
Iodine, μg/d	110^4^	152 (148, 156)	147 (144, 149)	145 (136, 154)	157 (149, 166)	146 (130, 163)

1 Values are means (95% CIs) unless otherwise indicated. Values in the same row without a common superscript letter are significantly different, *P* < 0.05; rows with no letters have no significant differences between values (ANOVA with Bonferroni correction). Picky eating scores (longitudinal) were assessed with the use of a questionnaire and were defined as follows: 0, not choosy; 1, quite choosy; and 2, very choosy. The responses for singletons were scored 0–2. The overall prevalence of picky eating for scores of children aged 2, 3, 4.5, and 5.5 y was calculated from the number of cases that reported a picky eating score of 2 at ≥2 time points. Early-onset picky eating was defined as the first report of picky eating (score of 2) occurring at the age of 2 or 3 y; late-onset picky eating was defined as the first report of picky eating (score of 2) occurring at the age of 4.5 or 5.5 y. Persistent picky eating was defined as a score of 2 at the first and/or second time points and then a score of 2 at both the third and fourth time points. ALSPAC, Avon Longitudinal Study of Parents and Children; EAR, estimated average requirement; NE, niacin equivalent; RE, retinol equivalent; RNI, reference nutrient intake.

2 Intake above which will almost certainly be adequate (2 notional SDs above EAR).

3 From reference 38.

4 From reference 39.

5 From references 39 and 40 [United Kingdom guideline revised to <5% in 2015 (41)].

6 Carotene is in the form of β-carotene equivalents: sum of β-carotene and half the amounts of α-carotene and α- and β-cryptoxanthins.

### Longitudinally defined picky eating types and diet at 7.5 y: micronutrients

Mean intakes of carotene, vitamin D, iron, zinc, and selenium were all highest in the never group and lower in the late-onset (8–26% lower; all *P* < 0.05) and persistent groups (3–16% lower; all *P* < 0.05). The children who showed some signs of picky eating (low and nonpersistent picky eaters) also had lower mean intakes of iron and zinc than the never group (Table 3).

There was more evidence of low intakes of micronutrients at the age of 7.5 y (**Table 4**) than at 3.5 y (Table 2) both in nonpicky and picky eaters (percentage below RNI for zinc increasing from 47% to 69% in nonpicky children). Furthermore, children were generally more likely at later ages to have intakes below the LRNI, with inadequate retinol equivalents and zinc intakes being the most likely. Data from the FFQ are shown in Supplemental Table 3 and** Supplemental Table 4**.

**TABLE 4:** Comparisons of micronutrient intakes, assessed by food record at 7.5 y of age, with United Kingdom RNI and LRNI by longitudinally defined picky eating type at ages 2–5.5 y in a subsample of ALSPAC children^1^

		Children below RNI or LRNI, %				
				High		
				Early onset		
	Recommendation, EAR, or RNI	Never	Low	Nonpersistent	Persistent	Late onset
*n*		1350	3134	279	403	100
United Kingdom RNI^2^ (39)						
REs		28.7^a^	33.9^b^	36.6^a,b,c^	40.7^b,c^	52.0^c^
Niacin	6.6 mg NEs/4.2 MJ	13.0^a^	18.0^b^	17.9^a,b^	21.3^b^	21.0^a,b^
Iron	8.7 mg/d	57.3^a^	63.0^b^	64.5^a,b^	66.0^b^	66.0^a,b^
Zinc	7 mg/d	69.1^a^	75.2^b^	80.3^b^	81.4^b^	80.3^b^
Selenium	30 μg/d	4.8^a^	6.7^a,b^	9.7^b,c^	8.4^a,b,c^	14.0^c^
United Kingdom LNRI^3^ (39)						
REs	250 μg REs/d	2.8^a^	4.0^a,c^	4.7^a,b^	7.2^b^	9.0^b,c^
Niacin	4.4 mg NEs/4.2 MJ	1.0^a^	1.5^a,b^	1.8^a,b^	3.0^b^	4.0^a,b^
Iron	4.7 mg/d	1.1^a^	1.5^a,c^	3.2^a,c^	4.2^b^	5.0^b,c^
Zinc	4 mg/d	5.9^a^	8.8^b^	10.4^a,b,c^	9.2^a,b,c^	19.0^c^
Selenium	16 μg/d	0.2	0.4	0.4	0.2	1.0

1 Bonferroni correction (*z* tests) was used for column proportion comparisons. Values in the same row without a common superscript letter are significantly different, *P* < 0.05; rows with no letters have no significant differences between values. Picky eating scores (longitudinal) were assessed with the use of a questionnaire and were defined as follows: 0, not choosy; 1, quite choosy; and 2, very choosy. The responses for singletons were scored 0–2. The overall prevalence of picky eating for children aged 2, 3, 4.5, and 5.5 y was calculated from the number of cases that reported a picky eating score of 2 at ≥2 time points. Early-onset picky eating was defined as the first report of picky eating (score of 2) occurring at the age of 2 or 3 y; late-onset picky eating was defined as the first report of picky eating (score of 2) occurring at the age of 4.5 or 5.5 y. Persistent picky eating was defined as a score of 2 at the first and/or second time points and then a score of 2 at both the third and fourth time points. Vitamin D was not included because the recommended daily intake is 0 μg/d. ALSPAC, Avon Longitudinal Study of Parents and Children; EAR, estimated average requirement; LRNI, lower reference nutrient intake; NE, niacin equivalent; PE, picky eating; RNI, reference nutrient intake.

2 Intake above which will almost certainly be adequate (2 notional SDs above EAR).

3 Intake below which will almost certainly be inadequate for most individuals (2 notional SDs below EAR).

### Food group differences

Differences in the amounts of food groups consumed between the picky eating groups at 3.5 y were found for meat, fish, vegetables, fruits, and milk (**Table 5**) but not for other food groups (Supplemental Text). On average, picky eaters aged 3.5 y ate 32% less carcass (unprocessed) meat, 44% less fish, 36% less fruits, and 52% less vegetables than nonpicky eaters but drank 20% more milk by weight (all *P* < 0.05). Picky eaters were more likely to drink ≥600 g milk/d (23%) than nonpicky eaters (8%) and somewhat picky eaters (12%) (600 g milk is equivalent to 600 mL or just over 1 imperial pint or 2.5 American cups).

**TABLE 5:** Food group intakes assessed by food record at 3.5 y (cross-sectional) showing only those foods or drinks that differed by picky eating score at age 3 y in a subsample of ALSPAC children^1^

	Picky eating score		
Food group intakes, g/d	0	1	2
*n*	364	320	131
Total meat	61 (57, 65)^a^	52 (48, 56)^b^	50 (43, 57)^b^
Carcass meat^2^	38 (34, 43)^a^	29 (25, 32)^b^	26 (20, 32)^b^
Processed meat^3^	23 (20, 25)	23 (21, 26)	24 (20, 28)
Total fish	16 (14, 18)^a^	15 (13, 17)^a^	9 (7, 12)^b^
Total vegetables	52 (48, 57)^a^	42 (38, 46)^a^	25 (19, 31)^b^
Total fruits	72 (65, 78)^a^	68 (62, 75)^a,b^	46 (36, 56)^b^
Total milk^4^	325 (305, 344)^a^	347 (322, 372)^a,b^	390 (342, 437)^b^

1 Values are means (95% CIs) unless otherwise indicated. Values in the same row without a common superscript letter are significantly different, *P* < 0.05; rows with no letters have no significant differences between values (ANOVA with Bonferroni correction). Picky eating scores (cross-sectional) were assessed with the use of a questionnaire and were defined as follows: 0, not choosy; 1, quite choosy; and 2, very choosy. ALSPAC, Avon Longitudinal Study of Parents and Children.

2 Included lamb, pork, beef, poultry, liver, and kidney.

3 Included sausages, ham, bacon, burgers, meat pies, breaded poultry, salami, etc.

4 Included whole and semiskimmed milk, skimmed cow milk, other animal milks, soya milk, human milk, formula, and cream [100 g milk is equal to ∼100 mL (an American cup holds 236 mL)].

Most of the same food groups showed differences at 7.5 y when the longitudinally defined picky eating groups were compared (**Table 6**). The persistent and late-onset picky eaters showed the largest differences from the never-picky group in the amounts of these foods eaten (40% and 38% less carcass meat, 48% and 52% less vegetables, and 33% and 33% less fruits, respectively; all *P* < 0.05). Processed meat and total fish intake were not different between the groups, but some other foods showed differences not found in the younger children. Eggs and egg dishes, plain potatoes, and salad dressings, etc., were eaten in lower amounts by all the picky eating groups than the never group. The persistent group showed differences from the never group in the intake of some foods that did not differ in the other picky eating groups (buns, cakes, and pastries, 19% lower; sweet biscuits and cookies, 26% higher; chocolate confectionery, 33% higher). There was also a higher intake in the persistent group for total milk intake (14% greater than in the never group), although the proportion of children drinking ≥600 g/d was quite similar (9% in the persistent group and 6–8% in the other groups). There were higher intakes of carbonated sugar-sweetened soft drinks in most of the picky groups than in the never-picky group. FFQ data are presented in **Supplemental Tables 5** and** 6**.

**TABLE 6:** Food group intakes, assessed by food record at 7.5 y, showing only those foods and drinks that differed by longitudinal picky eating groups at ages 2–5.5 y in a subsample of ALSPAC children^1^

	Picky eating				
			High		
			Early onset		
Food group intakes, g/d	Never	Low	Nonpersistent	Persistent	Late onset
*n*	1350	3134	279	403	100
Total meat	85 (83, 88)^a^	78 (77, 80)^b^	77 (71, 83)^a,b^	62 (58, 66)^c^	66 (57, 74)^b,c^
Carcass meat^2^	45 (43, 47)^a^	40 (38, 41)^b^	36 (31, 41)^b,c^	27 (24, 30)^d^	28 (22, 34)^c,d^
Processed meat^3^	40 (39, 42)	38 (37, 39)	41 (37, 45)	35 (32, 38)	38 (32, 43)
Eggs and egg dishes	9 (8, 10)	7 (7, 8)^a^	7 (5, 8)^a^	5 (4, 7)^a^	4 (2, 6)^a^
Plain or mashed potatoes	34 (32, 36)^a^	30 (28, 31)^b^	25 (21, 29)^b,c^	21 (18, 24)^c^	22 (15, 28)^b,c^
Total vegetables	66 (64, 69)^a^	56 (54, 58)^b^	51 (46, 56)^b^	34 (30, 38)^c^	32 (24, 40)^c^
Total fruits	87 (83, 91)^a^	78 (75, 81)^b^	71 (62, 79)^b,c^	58 (52, 65)^c^	57 (45, 68)^b,c^
Salad dressing, barbecue sauce, etc.	13 (13, 14)^a^	11 (11, 12)^b^	10 (8, 12)^b,c^	8 (7, 9)^c^	8 (6, 10)^b,c^
Buns, cakes, and pastries	31 (29, 32)^a^	30 (29, 31)^a^	27 (23, 30) ^a b^	25 (22, 27)^b^	25 (20, 30)^a,b^
Total milk^4^	262 (252, 272)^a^	255 (248, 261)^a^	263 (239, 387)^a,b^	299 (277, 320)^b^	264 (217, 311)^a,b^
Sweet biscuits and cookies	19 (18, 20)^a^	21 (20, 21)^a^	22, (20, 24)^a,b^	24 (22, 26)^b^	22 (19, 26)^a,b^
Chocolate confectionery	15 (14, 16)^a^	16 (15, 17)^a^	17 (15, 19)^a,b^	20 (18, 22)^b^	18 (15. 22)^a,b^
Carbonated drinks with sugar	64 (58, 71)^a^	79 (74, 84)^b^	98 (79, 117)^b^	86 (72, 100)^b^	75 (48, 102)^a,b^

1 Values are means (95% CIs). Values in the same row without a common superscript letter are significantly different, *P* < 0.05; rows with no letters have no significant differences between values (ANOVA with Bonferroni correction). Picky eating scores (longitudinal) were assessed with the use of a questionnaire and were defined as follows: 0, not choosy; 1, quite choosy; and 2, very choosy. The responses for singletons were scored 0–2. The overall prevalence of picky eating for children aged 2, 3, 4.5, and 5.5 y was calculated from the number of cases that reported a picky eating score of 2 at ≥2 time points. Early-onset picky eating was defined as the first report of picky eating (score of 2) being at the age of 2 or 3 y; late-onset picky eating was defined as the first report of picky eating (score of 2) being at the age of 4.5 or 5.5 y. Persistent picky eating was defined as a score of 2 at the first and/or second time points and then a score of 2 at both the third and fourth time points. A list of other food groups compared are provided in Supplemental Text. ALSPAC, Avon Longitudinal Study of Parents and Children.

2 Included lamb, pork, beef, poultry, liver, and kidney.

3 Included sausages, ham, bacon, burgers, meat pies, breaded poultry, salami, etc.

4 Included whole and semiskimmed milk, skimmed cow milk, other animal milks, soya milk, human milk, formula, and cream [100 g milk equivalent to ∼100 mL (an American cup holds 236 mL)].

### Results of the FFQ data analysis

The FFQ data confirmed all findings from the FR data (Supplemental Tables 1–6). The FFQ was more likely to show differences in nutrient intakes between the picky eating groups (*P* < 0.05) than the FR. The FFQ results indicated a higher percentage of children below the LRNI for retinol equivalent, iron, and zinc intakes when comparing picky eaters with nonpicky eaters at 3 y. For all 3 nutrients, the FFQ data suggested a higher proportion of children below the LRNI than did the FR data. There were similar findings for the longitudinally defined picky eating groups. The food group data from the FFQ confirmed the findings from the FRs except for milk intake, for which there were no differences between the groups either at 3 y or when picky eating was defined longitudinally.

## DISCUSSION

We found that picky eating defined at 3 y of age and picky eating defined longitudinally between 2 and 5.5 y of age were associated with a slightly lower protein intake and lower intakes of some micronutrients, particularly carotene, iron, and zinc, compared with nonpicky eaters. There was no evidence of a compromise of energy intake in picky eaters, and mean energy and particularly protein intakes were high in all groups relative to recommendations. In the longitudinal data at 7.5 y of age there was evidence of higher mean intakes of free sugars in the picky eating groups, and all groups had mean intakes much higher than the recommended maximum. For the micronutrients in older-aged children, the proportion of children with intakes below the LRNI was slightly higher in the picky eaters than the nonpicky eaters. There was a greater percentage of children with low nutrient intakes at 7.5 y than at 3.5 y of age whether picky eaters or not, suggesting that children were at greater risk of nutrient deficiency in older ages. The nutrient differences were explained by the lower intakes of meat, vegetables, and fruits in picky eaters compared with nonpicky eaters both cross-sectionally and longitudinally; in the older picky-eating children there were higher intakes of sugary foods and drinks. Parents should be supported in increasing intakes of nutrient-rich foods in children who are picky about food. All children should be encouraged to consume a range of foods from vegetables and fruits and, to a lesser extent, protein food groups and to consume fewer sugary foods and drinks to enhance their intakes of essential nutrients, particularly iron, zinc, and carotene, without increasing their overall intake of energy. Parents are important role models for their children, and eating well-balanced meals together is an excellent way to improve children’s diets (42).

This study is unique in investigating diet in relation to the picky eating status of children from 2 viewpoints: current diet in picky eaters aged 3 y and later diet in children who continued to be picky eaters over time. Both analyses made comparisons with children who were not picky eaters. In children aged 3 y, we assessed diet within 4–5 mo of defining their picky eating status. Differences were found between the picky eating groups for protein and some micronutrients, and there were differences in the amounts consumed of the key food groups: meat, fish, vegetables, and fruits. The diet assessed at the age of 7 y was compared between groups of children whose picky eating status had been defined ≥2 y earlier, between the ages of 2 and 5.5 y; despite this, the nutrients and foods affected were extremely similar to those found in children aged 3 y for the current diet. In this case, 2 of the picky eating groups showed much larger differences from the never-picky group: the persistent and late-onset (>3 y) groups. There was evidence in the persistent picky eaters of higher mean intakes of some sugary foods, in line with the overall higher mean free sugar intake found in picky compared with nonpicky groups at the age of 7 y. Children whose parents indicated that they were somewhat picky (quite choosy) or who were only picky at or before 3 y of age tended to have intermediate intakes of nutrients and food groups. These results suggest that children can be compromised in their intake of certain nutrients whether picky eaters or not, but children who are picky at any age are slightly more likely than children who are never picky to have intakes that are out of line with recommendations.

In general, our results confirm previous studies that have suggested that picky eating has little effect on macronutrient intakes, particularly in preschool-aged children (14, 43). As in this study, a Chinese study (16) reported that protein intakes were lower in picky eaters than nonpicky eaters, but again, intakes were adequate. In a study of Canadian children aged 2.5, 3.5, and 4.5 y (26), children who were picky eaters consumed less energy, fat, and protein than children who were never reported as being a picky eater, but energy intakes in all groups were adequate and differences were small (322 kJ/d for differences between never reported compared with reported at all 3 ages). Protein intakes were also adequate. It is possible that the effects on macronutrient intakes, particularly energy, may be more pronounced in older picky eaters, in whom the behavior is likely to be more established and persistent (15, 24). We found higher intakes of free sugars in the picky eating groups in children aged 7 y in this study; intakes of free sugars in picky eaters have not previously been reported to our knowledge.

Our results also confirm the findings of previous studies that have suggested that picky eating may be associated with deficits in some micronutrients (4, 43). For example, in a study of diets in US children aged 3–7 y (43), magnesium, calcium, and vitamin A, C, D, and B-12 intakes exceeded US recommendations, but those of zinc and vitamins D and E were <100%. Mean nutrient intakes for picky eaters, however, were not significantly different from those of nonpicky eaters at any time point, emphasizing the importance of including a comparison group. In this study, which did include a comparison group, the main differences between picky eating groups were for retinol equivalents (mainly carotene), iron, and zinc. More differences were apparent when comparing the FFQ-assessed diet between the groups, possibly because the FFQ results were based on the frequency of consumption with standard portion sizes, thus magnifying the importance of frequency, whereas FRs with specific descriptions of portion sizes for each food as eaten were more sensitive to individual variation in both frequency and portion size.

As in our study, other studies have found lower intakes of fruits, vegetables, and protein-rich foods such as meat and fish (44–47) or lower intakes of vegetables and whole grains (20, 24, 48) in picky compared with nonpicky eaters. We found some evidence in children aged 3 y that some picky eaters drank large volumes of milk, which is in line with the suggestion that excessive milk drinking may be associated with a reduced appetite and intake of other foods (49).

There are several strengths of this study: *1*) the definition of picky eating with the use of an unambiguous question about child choosiness that did not invite the parents to define picky eating for themselves; *2*) the inclusion of nonpicky comparison groups in both analyses; *3*) the use of FRs with household measures, a well-respected method for assessing diet in the main analysis rather than the less-precise FFQ method (50); *4*) the use of United Kingdom RNIs to assess the adequacy of the nutrient intakes; and *5*) the opportunity to assess picky eating over time. Limitations include the following: *1*) picky eating categorization was based on a single question and did not cover the full range of picky eating traits as defined in some other studies; *2*) identification of avoidant or restrictive food intake disorder (previously known as selective eating disorder) according to the *Diagnostic and Statistical Manual of Mental Disorders, 5th Edition* definition was beyond the scope of this study and not possible within the constraints of the data available; *3*) there was a relatively small number of children in some of the picky eating status groups; *4*) attrition and incomplete data collection were inevitable [60% of the 10% subsample provided FRs at 3.5 y and 54% of the original cohort provided them at 7.5 y (51)]; and *5*) the United Kingdom dietary guidelines do not include recommendations for some nutrients, such as carotene and vitamin E (39).

In conclusion, there was little evidence that picky eating resulted in compromised macronutrient intakes. However, mean intakes of some micronutrients (retinol equivalents, zinc, and iron) were low in all groups of children and slightly lower in children with picky eating than those without in both cross-sectionally and longitudinally defined groups. A slightly higher proportion of picky eaters had intakes below the LRNI for these nutrients compared with nonpicky eaters. The main food group differences between picky and nonpicky children at both ages were in the lower amounts of meat, vegetables, and fruits they consumed; however, in the older children, picky eaters also consumed more of some sugary foods and drinks than nonpicky eaters. Emphasis should be placed on allaying parental concerns about their picky children being prone to inadequate nutrient intakes and on encouraging all parents to gradually extend the diets of their children to include more nutrient-rich items from the compromised food groups, especially fruits and vegetables, and less of the nutrient-poor sugary foods.

## Footnotes

7 Abbreviations used: ALSPAC, Avon Longitudinal Study of Parents and Children; FFQ, food-frequency questionnaire; FR, food record; LRNI, lower reference nutrient intake; RNI, reference nutrient intake.
